# Social Determinants of Health/Health Equity and Hypertension Prevalence Among Mississippi Adults

**DOI:** 10.3390/ijerph23060705

**Published:** 2026-05-26

**Authors:** Vincent L. Mendy, Luressie Jones-Lee, Nagasi Weldu Tsegay, Tawandra Rowell-Cunsolo, Byambaa Enkhmaa

**Affiliations:** 1 Department of Epidemiology and Biostatistics, College of Health Sciences, Jackson State University, 350 W. Woodrow Wilson Dr, Suite 2300, Jackson, MS 39217, USA; luressie.m.jones-lee@students.jsums.edu (L.J.-L.); negasi.tsegaymd@gmail.com (N.W.T.); 2 Sandra Rosenbaum School of Social Work, University of Wisconsin-Madison, Madison, WI 53706, USA; rowellcunsol@wisc.edu; 3 Department of Internal Medicine, School of Medicine, University of California, Davis, CA 95616, USA; ebyambaa@ucdavis.edu

**Keywords:** behavioral risk factor surveillance system, hypertension, high blood pressure, health equity, social determinants of health, Mississippi

## Abstract

**Highlights:**

**Public health relevance—How does this work relate to a public health issue?**

- Social determinates of health significantly influence hypertension prevalence.

**Public health significance—Why is this work of significance to public health?**

- Compared with Mississippi adults reporting no SDOH/HE risk factors, those experiencing one or four or more SDOH/HE risk factors had higher odds of hypertension.

**Public health implications—What are the key implications or messages for practitioners, policy makers and/or researchers in public health?**

- A higher number of SDOH/HE risk factors was associated with increased odds of hypertension prevalence.

**Abstract:**

More than one million adults in Mississippi have hypertension. We examined the association between a summary measure of social determinants of health (SDOH) and health equity (SDOH/HE) and hypertension prevalence among Mississippi adults. Using data from 3867 respondents to the 2023 Mississippi Behavioral Risk Factor Surveillance System SDOH/HE Module, we performed multivariable logistic regression analyses to examine the association between hypertension prevalence and a summary SDOH/HE measure. The SDOH/HE summary measure score was created using responses to 10 questions assessing employment/economic and housing stability, receipt of food assistance, access to quality food and transportation, utilities security, social isolation, social and emotional support, life satisfaction, and mental well-being of the participants. The likelihood of experiencing 0, 1, 2, 3, or 4 or more SDOH/HE risk factors was 38.6, 22.9%, 13.7%, 7.9%, and 16.8%, respectively. Mississippi adults experiencing one (adjusted odds ratio, AOR, 1.47) and four or more (AOR, 2.62) of SDOH/HE risk factors had higher odds of hypertension compared to those experiencing no SDOH/HE risk factors. Associations between presence of two or three SDOH/HE risk factors and hypertension were not significant. Mississippi adults with one and at least four SDOH/HE risk factors had significantly higher odds of hypertension risk than those with no SDOH/HE risk factors. These findings highlight an association between SDOH/HE factors and the burden of hypertension and underscore the need for targeted inventions among Mississippi adults with multiple SDOH/HE risk factors.

## 1. Introduction

Hypertension is a significant risk factor for cardiovascular disease, the leading cause of death in Mississippi [1], and is associated with premature death and all-cause mortality [2]. In 2023, Mississippi’s (65.8/100,000) hypertension age-adjusted death rate was double that of the national rate (31.9/100,000) [1]. Disparities in hypertension prevalence are well documented [2]; in 2023, nationally, Mississippi residents were about 1.4 times more likely to have hypertension compared with the national average [3]. In the Jackson Heart Study, which is comprised of African American adults in the Jackson, Mississippi, Metropolitan area, participants were more likely to have persistent blood pressure control if they were less than 65 years, women, and had a family income higher than $25,000 [4]. Among Mississippi adults aged 45 years or older, the overall age-adjusted hypertension death rate increased by 55.4% from 2000 to 2018 with a significant average annual increase of 3.0% [5]. Additionally, among Mississippi adults 18 years or older, the prevalence of hypertension significantly increased from 2013 (40.2%) to 2021 (43.9%) [6].

Social determinants of health (SDOH), including conditions such as safe housing, transportation, neighborhood environments, access to nutritious foods, experiences with discrimination, and health care access, significantly influence health, well-being and quality of life [7,8]. Growing evidence suggests that SDOH play a crucial role in shaping access to care and driving disparities in hypertension prevalence [9]. Because SDOH risk factors or experiences are highly interrelated, a composite SDOH measure, rather than evaluating each factor in isolation, could provide a more comprehensive understanding of the overall contribution to health disparities [10]. This approach may better elucidate the complex and overlapping conditions that shape health outcomes and may help identify Mississippians at greatest risk [11]. The rationale for this study is based on the need for Mississippi-specific evidence to inform public health intentions aimed at reducing hypertension-related disparities. Although prior research has identified associations between SDOH and hypertension prevalence, statewide data examining summary measures of SDOH/health equity (HE) and hypertension prevalence among Mississippi adults remain limited. Understanding these associations is important for developing targeted interventions that address not only clinical risk factors but also the broader social structural conditions contributing to hypertension burden in the state [12]. To address this gap, we examined associations between a summary measure of SDOH/HE and hypertension prevalence among Mississippi adults.

## 2. Materials and Methods

We analyzed data from the 2023 Mississippi Behavioral Risk Factor Surveillance System (BRFSS), which included the SDOH/Health Equity (HE) Module. Detailed information about the BRFSS is available from the Centers for Disease Control and Prevention BRFSS website (www.cdc.gov/brfss/, accessed on 12 February 2025). Briefly, BRFSS is a state-based landline and cellular telephone survey of noninstitutionalized adults 18 years or older that collects self-reported information on behavioral risk factors, access to and use of health care services, and chronic conditions. The survey was conducted in all 50 states, the District of Columbia and three U.S. territories (Puerto Rico, Guam and the U.S. Virgin Islands). BRFSS data provide reliable and valid estimates of health risk factors [13]. Post-stratification weighting is applied to account for nonresponse, undercoverage, and unequal probability selection of populations [13]. The BRFSS was approved by the human research review board at each state’s department of health. Analyses were restricted to respondents who self-identified as either non-Hispanic Black or non-Hispanic white (n = 3867; weighted n = 2,108,412). This study was determined to be exempt by the Jackson State University institutional review board.

### 2.1. Hypertension

The following question was used to measure hypertension: “Have you ever been told by a doctor, nurse or other health professional that you have high blood pressure?” and coded as yes or no.

#### The Social Determinants of Health and Health Equity (SDOH/HE) Module

We used responses to 10 questions from the 2023 SDOH/HE module. These questions, based on the Center for Medicare and Medicaid Innovation Social Needs Assessment Tool [10], asked about employment/economic stability (*In the past 12 months have you lost employment or had hours reduced?*), receiving food stamps or Supplemental Nutrition Assistance Program (SNAP) (*During the past 12 months, have you received food stamps, also called SNAP, the Supplemental Nutrition Assistance Program on an EBT card?*), housing stability and quality (*During the last 12 months, was there a time when you were not able to pay your mortgage, rent or utility bills?*), food security (*During the past 12 months how often did the food that you bought not last, and you didn’t have money to get more?*), transportation access (*During the past 12 months has a lack of reliable transportation kept you from medical appointments, meetings, work, or from getting things needed for daily living?*), utilities security (*During the last 12 months was there a time when an electric, gas, oil, or water company threatened to shut off services?*), social isolation (*How often do you feel lonely?*), social and emotional support (*How often do you get the social and emotional support that you need?*), life satisfaction (*In general, how satisfied are you with your life?*), and mental well-being (*Stress means a situation in which a person feels tense, restless, nervous or anxious or is unable to sleep at night because their mind is troubled all the time. Within the last 30 days, how often have you felt this kind of stress?*) [10].

We developed a summary SDOH/HE measure by coding the responses to each SDOH/HE item as 0 (minimal/no experience) or 1 (experience of the SDOH/HE risk factor) and summing all scores. The total SDOH/HE scores ranged from 0 to 10; and the respondents were categorized as having 0, 1, 2, 3, or ≥4 risk factors [10].

### 2.2. Statistical Analyses

Logistic regression analyses were used to estimate adjusted odds ratios (AORs) and 95% confidence intervals (CIs) for the association between the summary SDOH/HE measure and hypertension prevalence. Covariates included age, race, sex, education, income, smoking, exercise, insurance, and body mass index. All statistical analyses were performed using SAS, Version 9.4 (SAS Institute, Inc., Cary, NC, USA), with adjustment for the complex sample design.

## 3. Results

Of the respondents, a quarter (25.3%) were 65 years or older, more than one third (38.2%) were non-Hispanic Black, more than half had a high school education or higher (55.2%) and were women (52.4%), and 50.8% had an annual household income of $50,000 or more. Hypertension prevalence was 46.0%, and the likelihood of experiencing 0, 1, 2, 3, or 4 or more SDOH/HE risk factors was 38.6% (95% CI, 36.5–40.8), 22.9% (95% CI, 21.0–24.9), 13.7% (95% CI, 12.1–15.3), 7.9% (95% CI, 6.6–9.2), and 16.8% (95%, 15.1–18.5), respectively.

Mississippi adults experiencing one (AOR, 1.47; 95% CI, 1.03–2.10) or four or more (AOR, 2.62; 95% CI, 1.68–4.08) SDOH/HE risk factors had higher odds of hypertension compared to those experiencing no SDOH/HE risk factors (Table 1).

## 4. Discussion

Mississippi adults who reported experiencing one and four or more SDOH/HE risk factors had significantly higher odds of hypertension than those experiencing no SDOH/HE risk factors. The results suggest that SDOH are associated with elevated rates of hypertension-related disparities among Mississippi adults, which is consistent with the findings from national surveys. For example, in a study of 49,791 adults from the 1999–2018 National Health and Nutrition Examination Surveys, researchers identified a significant dose–response association between the number of adverse SDOH and hypertension [14]. In a cardio-oncology prospective cohort study of 381 participants, high SDOH scores resulted in a 77% increased risk for uncontrolled hypertension [15]. Similarly, The REasons for Geographic And Racial Differences in Stroke (REGARDS) Study participants (n = 14,803) with more adverse SDOH had a higher prevalence of uncontrolled blood pressure [16].

The lack of statistically significant association between two or three SDOH/HE risk factors and hypertension may be related to the relatively small number of respondents with multiple risk factors and therefore warrants further investigation.

Overall, these findings demonstrate the importance of evaluating the association between SDOH/HE burden and hypertension. Mississippi adults could benefit from utilizing more holistic measures of SDOH/HE to inform screening, prevention and hypertension management strategies in the state. The cumulative effect of multiple SDOH/HE risk factors underscores the need for integrated approaches to reduce hypertension risk among Mississippi adults.

A composite measure of SDOH/HE may be an effective tool for establishing priorities to pursue when addressing persistent hypertension-associated health disparities [16]. More comprehensive measures are crucial for informing treatment needs among hypertensive adults and identifying targeted interventions to reduce health inequities [12].

## 5. Limitations

The findings are subject to some limitations. First, the BRFSS data on SDOH/HE measures and hypertension were self-reported and therefore subject to recall and social desirability bias [17]. Second, the analysis of SDOH/HE Module data was limited to selected SDOH/HE items available in the dataset. Finally, because the study used a cross-sectional design, the observed associations cannot be interpreted as causal relationships.

## 6. Conclusions

A summary measure of SDOH/HE risk factors may help identify populations at higher risk for hypertension disparities among adults in Mississippi and support the development of targeted interventions. Understating the cumulative impact of social and economic disadvantage on hypertension can inform public health strategies aimed at improving cardiovascular health outcomes in Mississippi [11]. Futures studies should examine the longitudinal association between SDOH/HE risk factors and hypertension to better understand the causal and temporal associations.

## Figures and Tables

**Table 1:** Associations between a summary measure of SDOH/HE and hypertension prevalence among Mississippi adults, BRFSS, 2023.

Characteristics	Hypertension Prevalence			
	Model I		Model II	
	OR ^a^	95% CI	AOR ^b^	95% CI
Number of SDOH/HE				
0 (Referent)	1.00		1.00	
1	1.12	0.88–1.43	1.47	1.03–2.10
2	1.15	0.86–1.70	1.53	0.92–2.29
3	1.12	0.77–1.62	1.68	0.99–2.84
4+	1.37	1.05–1.79	2.62	1.68–4.08

AOR, adjusted odds ratio; BRFSS, Behavioral Risk Factor Surveillance System; CI, confidence interval; OR, odds ratio; SODH/HE, social determinant of health and health equity. ^a^ Unadjusted. ^b^ Adjusted for age, race, sex education, income, smoking, exercise, insurance, body mass index.

## Data Availability

Data used in study are publicly available at (https://www.cdc.gov/brfss/data_tools.htm, accessed on 12 February 2025).
